# Primary giant hepatic neuroendocrine neoplasms in a young adult: case report and literature review

**DOI:** 10.3389/fonc.2025.1555736

**Published:** 2025-07-01

**Authors:** Xue Luo, Xingxing Bie, Na Luo, Wenting Huang

**Affiliations:** ^1^ Department of Pathology, National Cancer Center, National Clinical Research Center for Cancer, Cancer Hospital and Shenzhen Hospital, Chinese Academy of Medical Sciences and Peking Union Medical College, Shenzhen, Guangdong, China; ^2^ Department of Thoracic Surgery, The Second Affiliated Hospital of Shenzhen University, Shenzhen, Guangdong, China

**Keywords:** liver, neuroendocrine neoplasms, synaptophysin, chromogranin, surgery

## Abstract

Primary hepatic neuroendocrine neoplasms (PHNENs) are a rare type of neuroendocrine tumors originating in the liver. These tumors are characterized by non-specific clinical symptoms and atypical imaging features, making differentiation from other primary hepatic masses, such as hepatocellular carcinoma (HCC) and parasitic lesions, challenging. The diagnosis of PHNENs is based on characteristic histological features associated with this condition and results from immunohistochemistry assays. Here, we report on a case of a 22-year-old female presenting with a large hepatic neoplastic lesion. Computed tomography (CT) imaging results revealed a highly vascularized mass with clear boundaries located in the right lobe of the liver, suggesting a diagnosis of HCC. The patient underwent a fine-needle aspiration biopsy and subsequent complete surgical resection of the tumor. Results from both the fine-needle aspiration and histology of the surgically resected specimen showed that the tumor cells were arranged in a solid structure with a trabecular pattern. The tumor cells exhibited positive expressions for the epithelial cell markers AE1/AE3, along with the neuroendocrine markers, synaptophysin (Syn), chromogranin (CgA), and CD56 as based on results from immunohistochemical staining. The Ki-67 proliferation index was > 20%, and the mitotic count was > 20 per 2 square millimeters, leading to a final diagnosis of a hepatic neuroendocrine neoplasms, Grade 3 (G3). PHNENs are extremely rare, and, to our knowledge, there have been no reports in the literature of adolescents or young adults diagnosed with the G3-type.

## 1 Introduction

Neuroendocrine tumors (NETs), a heterogeneous group of tumors originating from neuroendocrine cells, are commonly located in organs such as the gastrointestinal tract, appendix, and lungs. The current prevalence of NETs is approximately 170,000, and the incidence rate has increased by 5 to 6 times over the past 40 years (1). The diagnosis of NETs is challenging and is closely related to the primary site of occurrence, severity of symptoms, and proliferative activity of the tumor (2). Among patients with NETs, the lungs and bronchi are the most common sites for these tumors, with the gastrointestinal tract and pancreas also showing relatively high rate of incidence (3). However, primary neuroendocrine tumors of the liver are extremely rare. NETs have an insidious display of onset, and due to their slow growth, are often only identified at advanced stages. The majority of NETs are found as a result of their metastases to other organs (4), with the liver being one of the most common sites for this metastasis. The liver is the largest internal organ in the human body and exerts an essential role, in sustaining life and overall health. It is involved in synthesizing and secreting bile, which promotes digestion and absorption in the body, the metabolism of proteins and other substances, as well as detoxification of harmful substances. However, the presence of PHNENs within the liver are rarely reported in the literature, with the majority of neuroendocrine tumors being metastatic to the liver (5, 6). The clinical and pathological features of PHNENs include a lack of specific clinical symptoms. This characteristic makes an early diagnosis of PHNENs challenging and often leads to the misdiagnosis as primary HCC (7). The diagnosis of PHNENs is based on two stringent criteria: 1) the liver tumor must exhibit neuroendocrine characteristics and 2) the exclusion of metastatic neuroendocrine tumors from outside the liver (8). The specific histological types along with treatment approaches significantly affect the prognosis for patients with PHNENs, which have a lower survival rate as compared with that of pancreatic neuroendocrine tumors (9). Here, we report a case of PHNENs within a young woman, as initially suspected from results of Positron Emission Tomography/Computed Tomography (PET-CT) revealing a large hepatic mass. Histologically, this mass exhibited significant morphological features of high-grade neuroendocrine tumors and characteristics of neuroendocrine marker expressions. To our knowledge, this has not been previously documented in the literature.

## 2 Materials and methods

### 2.1 Histopathologic and immunohistochemical examination

This case involves a patient from our hospital who underwent a fine-needle biopsy in January 2024, and subsequently had a liver tumor resection after six months of treatment. The surgically excised tissue specimens were fixed in a 10% neutral buffered formalin solution, and after a series of procedures including sampling and dehydration, 4μm sections were prepared to complete the preparation of pathological tissue sections. Subsequently, the Ventana automatic immunostaining method was used for pre-treatment of anti-Syn (ZGB-BIO, China), anti-CgA (Roche, Switzerland), and anti-CD56(ZGB-BIO, China) antibodies. All samples were processed using the relevant equipment according to the product instructions and observed under a microscope. Two professional pathologists assessed the tissue sections without knowledge of the related clinical data, and provided a comprehensive score for the intensity of tissue staining and the percentage of positive cells.

## 3 Case presentation

**Figure 1:**
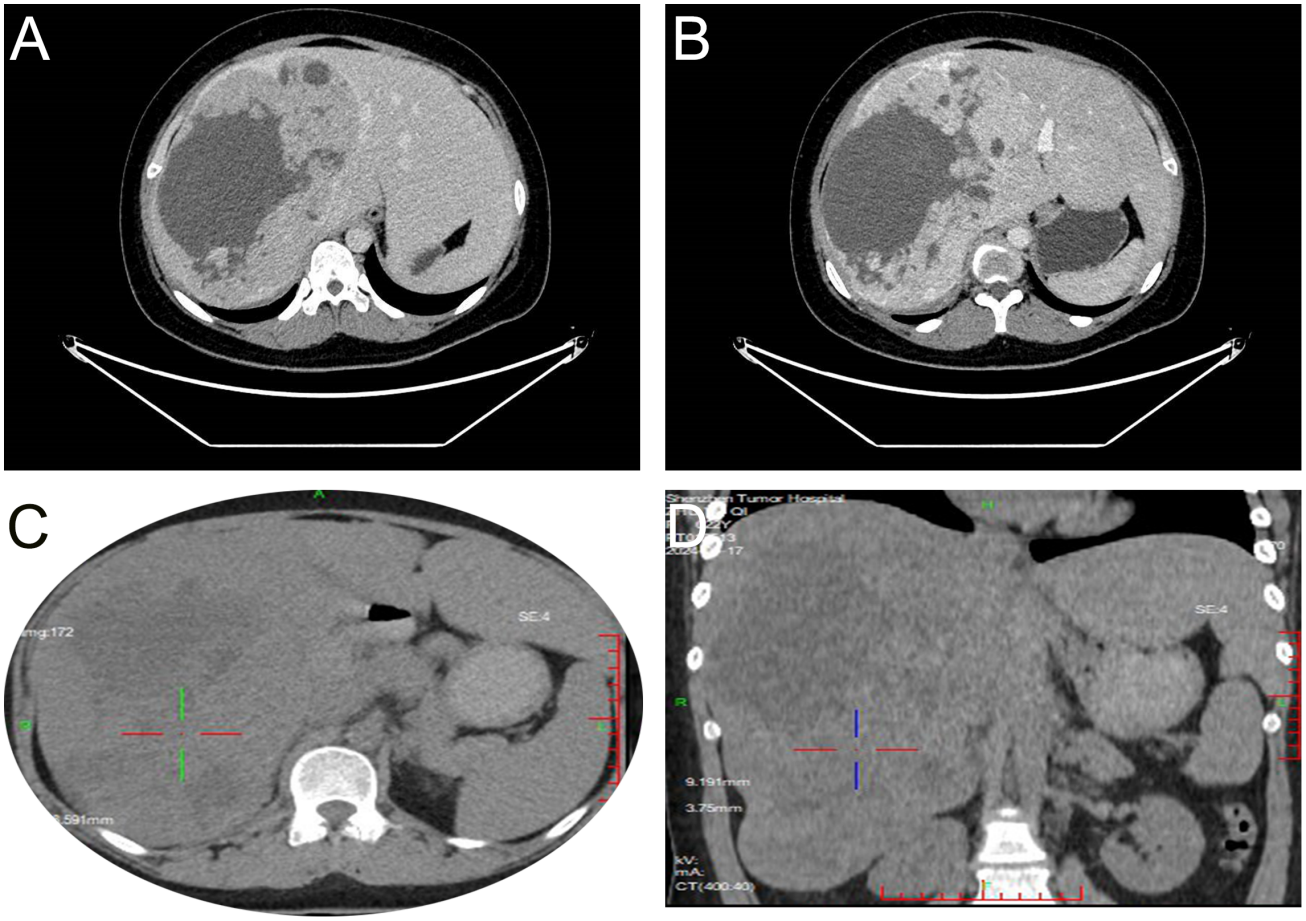
Imaging results. CT shows the tumor tissue located in the right lobe of the liver, with clear boundaries and mixed slightly low-density appearance with multiple patchy low-density shadows and a small amount of calcification (A). After enhancement, the arterial phase shows unevenly pronounced enhancement (B). PET-CT reveals a mixed-density mass in the right lobe of the liver, partially fused into a cluster, with unevenly increased uptake (1C, D).

**Figure 2:**
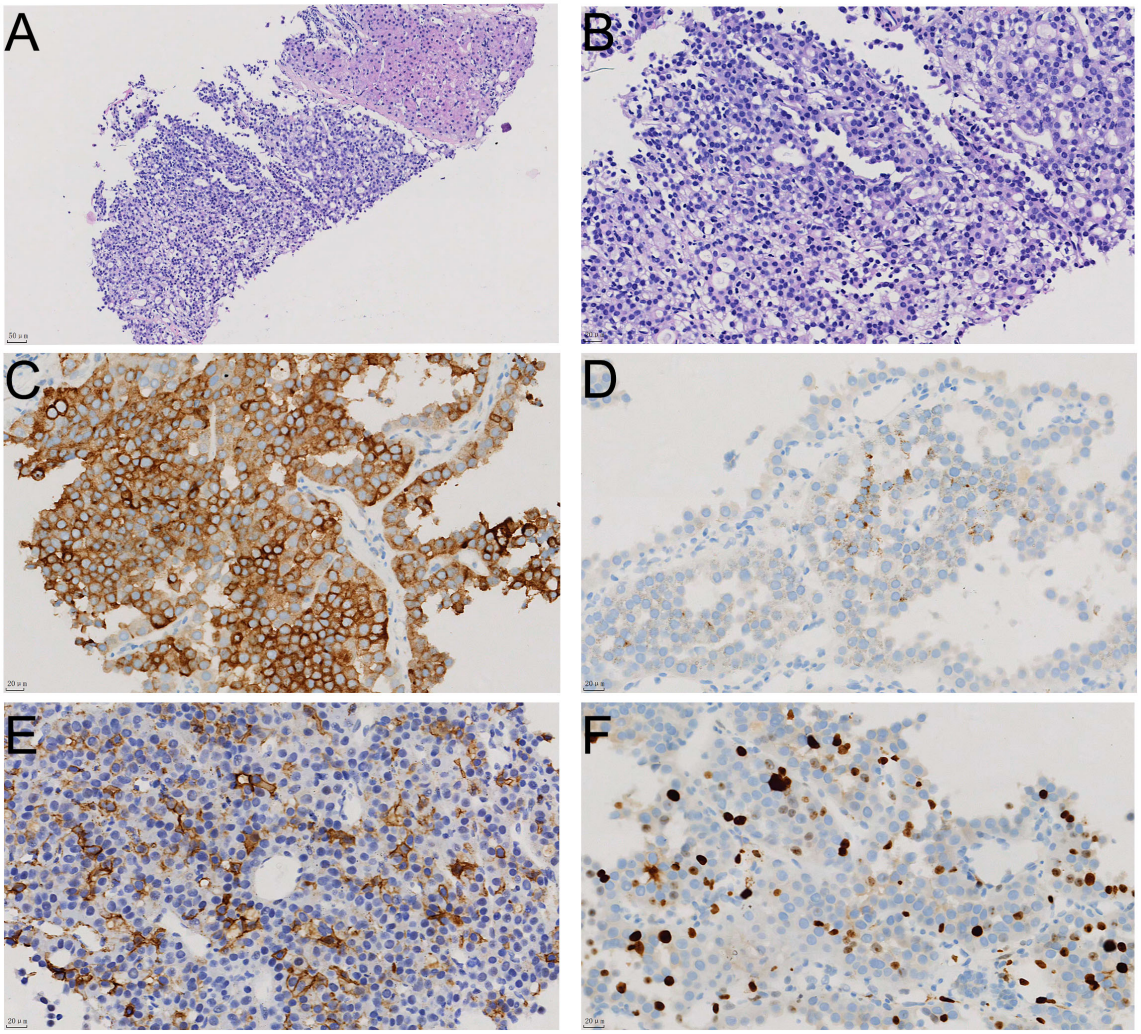
The histological appearance and immunohistochemical staining results of liver biopsy tissue. The relationship between tumor tissue and normal liver tissue is shown in HE staining **(A)**; HE staining reveals that the tumor tissue has a solid structure with cellular atypia **(B)**; immunohistochemical staining shows positive expression of neuroendocrine markers Syn **(C)**, CgA **(D)**, and CD56 **(E)** in tumor cells, with a Ki67 proliferation index of approximately 20% **(F)**.

During a routine physical examination at another hospital in December 2023 a liver mass was observed in a 22-year old female. Initially, this mass, was considered to be a hemangioma, and no specific treatment was administered. She was then referred to our hospital for further treatment. There was no history of chronic hepatitis in this patient and results from serological assays indicated that carcinoembryonic antigen, alpha-fetoprotein, carbohydrate antigen 19-9, and neuron-specific enolase, among other markers, were all within normal ranges. The CT scan, as performed at our hospital, revealed a large occupancy in the right lobe of the liver. This mass was 21×14 cm, with a rich blood supply, clear boundaries, and slightly mixed low-density shadows. There were multiple patchy and nodular low-density shadows inside, along with a small amount of calcification (**Figures 1A, B**). Such imaging results suggested a high possibility for HCC. A subsequent PET-CT revealed a significant enlargement of the liver volume, with a mixed-density mass in the right lobe, presenting as a confluent mass, with the largest cross-section measuring 21×14 cm. The mass protruded locally from the liver contour and compressed adjacent organs (**Figure 1C, 1D**). No definite occupancy lesions in other regions of the body were observed. An ultrasound-guided biopsy of the liver mass was performed in January 2024 with the resultant,histological finding revealing that the tumor exhibited a solid pattern, with round or oval nuclei and indistinct cell boundaries (**Figures 2A, B**). Immunohistochemical staining indicated that the tumor cells expressed Syn (**Figure 2C**), CgA(**Figure 2D**), and CD56(**Figure 2E**), along with a high proliferation index of Ki67(**Figure 2F**). These pathological results suggested a neuroendocrine tumor, of at least Grade2 (G2), and, with the findings of 9 mitotic figures/2 square millimeters and a high Ki67 proliferation index, the possibility of Grade3(G3) could not be excluded. Such neuroendocrine tumors in young adults are relatively rare. The attending physician assembled a multidisciplinary team (MDT) consisting of the Departments of Radiology, Pathology, Hepatobiliary Surgery, Medical Oncology, Interventional Radiology, and Radiation Oncology. Following a comprehensive analysis and discussion, the MDT recommended initiating targeted therapy, followed by contrast-enhanced CT scans and tumor marker assessments every three months to evaluate treatment responses and detect any new metastases. A complete surgical resection of the mass would be considered as clinically indicated for further evaluation.

**Figure 3:**
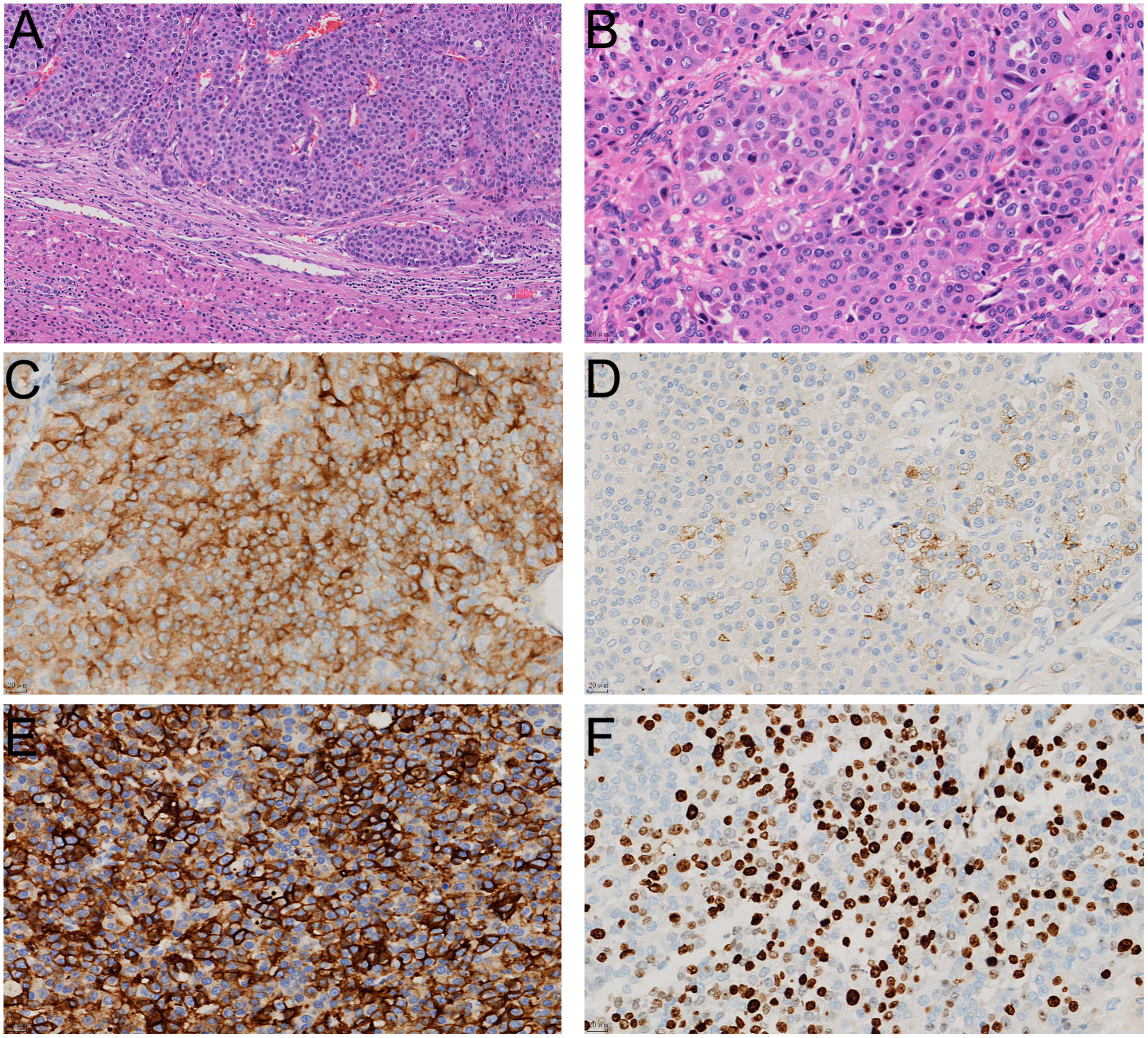
The histological appearance and immunohistochemical staining results of the surgically resected specimen. In HE staining, the tumor tissue is located within the liver parenchyma, exhibiting invasive growth **(A)**; HE staining reveals that the tumor tissue is solid and arranged in nest-like clusters, with cells showing significant atypia **(B)**; immunohistochemical staining shows positive expression of neuroendocrine markers Syn **(C)**, CgA **(D)**, and CD56 **(E)** in tumor cells, with a Ki67 proliferation index of approximately 30% **(F)**.

Following completion of this comprehensive diagnostic workups, the patient underwent six cycles of combination chemotherapy with tegafur capsules (60 mg/m²/day) and temozolomide (200 mg/m²/day) as administered over the period from February to June of 2024, with each cycle spanning 28 days. During the treatment period, no significant reductions in tumor size were observed, and the patient exhibited a heterogeneous elevation of somatostatin receptor expression. Following further clinical discussions of the MDT in July of 2024, the shared conclusion was to perform an extended right hepatectomy with cholecystectomy while the patient was under general anesthesia. The postoperative tumor specimen, which occupied the entire right lobe of the liver, was found to be grayish-white, cystic-solid, multinodular and hardened in texture. Results from the pathology examination revealed that the tumor was arranged in nest-like and trabecular patterns, with cellular atypia, eosinophilic cytoplasm, and prominent nucleoli in some areas (**Figures 3A, B**). Immunohistochemical staining showed similar results as before (**Figures 3C-E**), however, the Ki-67 (**Figure 3F**) proliferation index now achieved 30%. The final diagnosis was PHNENs, Grade 3. As of January 2025, the patient’s laboratory values have been normal, with no recurrence of the mass and a good prognosis.

## 4 Discussion and literature review

NETs are a heterogeneous group of rare epithelial tumors, with the liver being a common site for metastasies (5). The incidence of NETs has significantly increased over the past four decades, and they are now the second most prevalent gastrointestinal malignancies in the UK and USA (10). These patients often present with a metastatic disease, and the liver represents the most common site of metastasis for gastro-enteropancreatic NETs (11). The incidence of PHNETs is slightly higher in females(58.5%) than males(41.5%)and are more common in individuals over the age of 40 (12). The diagnosis of PHNENs primarily relies on results as obtained from biopsies of pathological tissue, with most PHNENs exhibiting a diffuse positivity for Syn and CD56 along with a rare focal expression of HepPar1 or glypican-3. In contrast, HCC, which can be misdiagnosed as PHNENs, rarely expresses Syn or CD56 but demonstrates a near-universal positivity for HepPar1 or glypican-3. Accordingly, immunohistochemical staining for these markers is critical to reliably differentiate HCC from PHNENs (13). Imaging also plays a crucial role in determining whether any accompanying metastatic tumors may be present in other body regions. In specific, magnetic resonance imaging (MRI) and PET-CT are becoming standard protocols for use in imaging of liver metastases. PHNENs exhibit distinct clinical and imaging characteristics. Notably, in patients with normal levels of liver tumor markers, suspicion for hepatic NENs should be raised if:1) significantly elevated NSE levels are present, 2), there is a presence of multiple intrahepatic masses with peripheral cyst/necrosis and similar rim arterial phase hyper-enhancement along with peripheral ‘washout’ in the venous portal system and 3)delayed phases on CT or MRI imaging are observed (7). Diagnosing PHNENs from imaging of liver parasitic diseases can be challenging, as they may share overlapping imaging features. In most cases, it is necessary to combine clinical manifestations with epidemiological history, and, in some cases, a liver biopsy to assess pathogenic microorganisms as a means to further clarify the diagnosis (14). Management of NENs can be challenging and depends on the primary tumor site, symptom severity, and proliferative activity. Surgical resection remains the preferred curative option, but many patients may not meet the criteria for surgery due to multifocal lobar involvement (15). Systemic medical therapy has been used for managing tumor burden and symptoms resulting from NETs, with somatostatin analogues being the primary treatment for carcinoid syndrome (16). The diagnosis of PHNENs must strictly meet two important criteria: 1) positive immunohistochemical staining for neuroendocrine markers and 2) an absence of any other organ space-occupying lesions outside the liver. The World Health Organization (WHO) revised its pathological grading system for neuroendocrine tumors in 2010, but PHNENs were not included in this revision, indicating a need for further validation of the grading system as applied to PHNENs (17). Positive rates for Syn have been reported to be at 55%, and 89.1%for CgA (12). To date, only 150 cases have been reported in the literature, of which only a portion were primary to the liver (18). The following description includes a summary of some of the clinical characteristics and immunohistochemical staining profiles associated with PHNENs (**Table 1**).

**Table 1:** The clinical and pathological features of PHNENs.

Age(y)	Gender	Size(cm)	Localization	Positive immunomarkers	Ki67	Grade	Reference
64	female	4.0x3.5	S5/8 segment	Syn+, CgA+, NSE+	25%	G3	(19)
74	female	2.5x2.4	S7/8 segment	Syn+, CgA+, NSE+	15%	G2	(19)
51	female	14.7x11	S3 segment	Syn+,CgA+,NSE+,CD56+	2%	G2	(20)
34	male	12x10	Right lobe	Syn+,CgA+	+	NA	(21)
52	female	16.2	Left lobe	Syn+,CgA+	24%	G3	(22)
45	female	8x3	Both liver lobes	Syn+,CgA+	10%	G2	(23)
60	male	4x3.5	S6 segment	Syn+,CD56+	5%	G2	(24)
22	female	6.6x4.2	S6 segment	Syn+,CgA+, CD56+	1-2%	G1	(25)
38	male	9.6x8.4	Right liver lobe	Syn+,CgA+, CD56+	3-5%	G2	(26)
72	male	NA	Multiple masses	Syn+,CgA+, CD56+	80%	G3	(27)
78	male	NA	Left lobe	Syn+,CgA+, CD56+	90%	G3	(28)
55	male	18.3x15.8	Right lobe	Syn+, CD56+	10%	G2	(29)
82	male	6.0x5.5	Right lobe	Syn+, CD56+	70%	G3	(30)
52	female	7.0x5.5	Liver	Syn+,CgA+	15%	G2	(31)
27	female	24x14	Right lobe	Syn+,CgA+, CD56+	15%	G2	(32)
84	female	0.8	S5 segment	Syn+,CgA+	80%	G3	(33)
41	female	8.2x7.4	Right lobe	Syn+, CD56+	5%	G2	(34)
73	male	3.0x2.6	S8 segment	Syn+, CD56+	30%	G3	(35)
72	female	3.0x2.5	S8 segment	NSE+	+	NA	(36)
64	female	3	S1 segment	Syn+,CgA+	6%	G2	(37)

NA (No available), +(positive).

Ki67 plays a significant role in the diagnosis of PHNENs. It provides a marker for cell proliferation, being expressed in the G1, S, G2, and M phases of the cell cycle, and rapidly catabolized at the end of the M phase, making it undetectable in G0 and early G1 cells (38). In the context of PHNENs, the Ki67 proliferative index serves as a well-documented and accepted diagnostic and prognostic parameter, and its evaluation is mandatory for a comprehensive diagnostic work-up (39). With regard to molecular markers, there are reports indicating the presence of TP53 mutations in PHNENs (40). Although the paraneoplastic syndrome, carcinoid syndrome, can be associated with PHNENs, this is a relatively infrequent event with only two cases reported in the literature. Carcinoid syndrome is characterized by a variety of symptoms that are not directly attributable to tumor invasion, compression, or metastasis, but rather are the result of secretions of functional hormones or peptides by the tumor and/or may be related to immune cross-reactivity with the host tissue (41, 42). Surgical resection remains the first line of treatment for resectable lesions and can significantly improve survival outcomes (9). The patient in this case had only a mild elevation in blood pressure since the onset of the disease, without any other paraneoplastic syndrome symptoms, and no significant abnormalities in serological assay values. PHNENs typically occur in middle-aged and elderly women (43), but this case involved a young adult female with a large single tumor in her liver. The grading of this tumor was G3, and, to our knowledge, there have been no reports in the literature of a young adult PHNENs patient with this grade. Our patient experienced minimal adverse responses to her medical treatment and currently survives after the surgical intervention performed.

## 5 Conclusion

In summary, PHNENs are rare, slowly growing tumors originating from neuroendocrine cells of the liver. those with higher grades are associated with an increased potential for malignancy and pose diagnostic challenges. The identification of typical histopathological patterns and expressions of characteristic immunohistochemical markers are crucial for diagnosis. The presence of specific genetic marker alterations will require further investigation.

## Data Availability

The original contributions presented in the study are included in the article/supplementary material. Further inquiries can be directed to the corresponding author/s.
